# Evaluation of Anticonvulsant Activity of 80% Methanolic Root Bark Extract and Solvent Fractions of *Pentas schimperiana* (A. Rich.) Vatke (Rubiaceae) in Swiss Albino Mice

**DOI:** 10.1155/2021/6689879

**Published:** 2021-06-07

**Authors:** Nebeyi Fisseha, Workineh Shibeshi, Daniel Bisrat

**Affiliations:** ^1^Department of Pharmacy, Mizan Tepi University, Mizan Teferi, Ethiopia; ^2^Department of Pharmacology, School of Pharmacy, Addis Ababa University, Addis Ababa, Ethiopia; ^3^Department of Pharmacognosy, School of Pharmacy, Addis Ababa University, Addis Ababa, Ethiopia

## Abstract

**Background:**

Epilepsy is one of the most common serious neurological disorders, responsible for substantial morbidity and mortality due to limited efficacy and negative properties of antiepileptic drugs. Medicinal plants are believed to be an important source of new chemical substances with potential therapeutic effects. *Pentas schimperiana* (A. Rich.) Vatke is a medicinal plant used in Ethiopian traditional medicine for the treatment of epilepsy. However, it lacks scientific investigation on its anticonvulsant activity. Therefore, this study was conducted to evaluate the anticonvulsant activity of 80% methanol root bark extract and solvent fractions of *Pentas schimperiana* (A. Rich.) Vatke in mice.

**Methods:**

Anticonvulsant activity was evaluated by using the pentylenetetrazole and maximal electroshock-induced seizure test. The 80% methanolic root bark extract was subjected to successive fractionation with solvents differing polarity, i.e., chloroform, butanol, and water. The test groups received 100, 200, and 400 mg/kg bodyweight of extract and its solvent fractions.

**Result:**

The ME400 and BF400 at the higher dose exhibited a significant (*p* < 0.001) anticonvulsant effect in both the pentylenetetrazole and maximal electroshock-induced seizure test compared with control. However, chloroform fraction only showed a significant (*p* < 0.001) anticonvulsant effect in the PTZ-induced seizure test, while aqueous fraction had least anticonvulsant activity in both seizure-induced tests. Phytochemical screening of *Pentas schimperiana* (A. Rich.) Vatke root bark extract revealed the presence of alkaloids, saponins, flavonoids, phenols, steroids, terpenoids, and tannins.

**Conclusion:**

This study indicated that the plant has anticonvulsant activity and is considered as a potential source to develop a new antiepileptic drug.

## 1. Introduction

Epilepsy is one of the most common serious neurological disorders, responsible for substantial morbidity and mortality due to the seizures and the available medications. Around 50 million people in the world have epilepsy, and approximately 5% of the general population experience at least one seizure, excluding febrile seizures, at some time in their lives [1]. Currently, available antiepileptic drugs (AEDs) have limited efficacy, and negative properties limit their use and cause difficulties in patient management [2]. It is worthwhile to develop new AEDs with minimal adverse reaction and effective against drug resistance epilepsy [3].

Herbal treatment is also used for about 75–80% of the world population for primary healthcare, mainly in the developing countries due to better cultural acceptability, better compatibility with the human body, and lesser side effects. Considering the great reliance on traditional medicinal plants for treatment of diseases and the potential for drug discovery, it becomes relevant to search for potent, effective, and relatively safe plant medicines [4]. Many kinds of medicinal plants have been used in folk medicines to treat epilepsy [5]. *Pentas schimperiana* (A. Rich.) Vatke is one such plant that is used traditionally for treatment of epilepsy [6]. However, no experimental studies have been conducted in Ethiopia so far to confirm the medicinal value of claimed antiepileptic plant *Pentas schimperiana* (A. Rich.) Vatke. The present study was carried out to provide baseline information on the traditional claim of *Pentas schimperiana* (A. Rich.) Vatke for epilepsy.

*Pentas schimperiana* is shrubby and semiwoody herb which reaches up to 2 m high. The leaves are narrow at both ends, and the petiole is ranging up to nearly half inch. It has purplish black woody stems, and the corolla is tube funnel shaped and the fruits are 4–6 mm long. It occurs in altitude range between 1710 and 2350 m. Its habitat is thickest at stream side, marshy edges, and open ground. *Pentas schimperiana* (A. Rich.) Vatke is subspecies of *Pentas schimperiana* found in Ethiopia, while subspecies occidental is occurring in Cameroon [7]. In Ethiopia, it is widely distributed in the area of Adaa district [8], Wonago woreda [6], Gedo dry evergreen mountain forest [9], Odo-Bulu, and Demaro in Bale Region [10].

*Pentas schimperiana* (A. Rich.) Vatke is known by its vernacular name as Woinagrefet, also known as Dibexxo (Gideo) [6] and Moonyeer or Dasie (Afaan Oromo) [8] with local name. Its dry root bark powder mixed with water is taken orally for epilepsy in Wonago woreda. In addition, root bark coarse powder is mixed with water given orally for treating livestock mental problem [6]. The experimental studies showed that the decoctions of leaves were used for antioxidant and antidiabetic activities [7].

## 2. Materials and Methods

### 2.1. Drugs and Chemicals

Absolute methanol (Carlo Erba, France), chloroform (Loba Chemie, India), phenytoin (Brawn, India), sodium valproate (Elfin Drugs, India), n-butanol (Indenta, India), normal saline (Aculife Healthcare, India), pentylenetetrazole (Sigma-Aldrich Chemie, USA), distilled water (Ethiopian Pharmaceutical Manufacturing, Ethiopia), and electroconvulsiometer (Rolex, India).

### 2.2. Plant Collection and Authentication

The root bark of *P. schimperiana* (A. Rich.) Vatke was collected in January, 2017, from its natural habitat around Wonago woreda, Gedeo Zone, Southern Nations, Nationalities, and Peoples' Region (SNNPR). Identification and authentication of the plant specimens were performed by a taxonomist at the National Herbarium, College of Natural and Computational Sciences, Addis Ababa University, where a voucher specimen was kept there for future reference with voucher no. NF 001/2017.

### 2.3. Preparation of Extracts

Root bark of *Pentas schimperiana* (A. Rich.) Vatke was washed thoroughly with tap water and gauze to remove soil and air-dried under shade until the root bark became fully dried. The air-dried coarse powdered plant material was subjected to cold maceration extraction with 80% methanol for three consecutive days at room temperature. The resulting crude extract was separated from the marc with gauze and further filtered using Whatman filter paper No. 1 (Whatman plc, United Kingdom). The filtrates were combined, and the solvent was removed by evaporation under reduced pressure using rotavapor (Buchi Rotavapor R-200, Switzerland) with temperature not exceeding 40°C. The extract was then freeze-dried using a lyophilizer (Operan, Korea Vacuum Limited, Korea). Finally, the concentrated extract was transferred into vials and kept at −20°C until use.

Then, extract was subjected to successive fractionation with solvents differing polarity, i. e., chloroform, butanol, and water. The crude extract was suspended in distilled water under a separatory funnel, and the suspension was shaken with chloroform added. Two layers were formed, and chloroform fraction was obtained by separation. The process was continued until the solution became clear. The aqueous layer was then shaken with butanol to obtain the butanol fraction. The butanol and chloroform filtrate was concentrated in a rotavapor at 40°C to obtain the butanol and chloroform fraction. The remaining aqueous residue was frozen in a deep freezer and then freeze-dried with a lyophilizer. At last, the fractions were kept in an amber glass bottle and stored in a refrigerator.

### 2.4. Animals

Healthy Swiss albino mice, 20–30g, aged 8–12 weeks, female mice for the acute oral toxicity test and male mice for the anticonvulsant activity study, were purchased from Ethiopian Public Health Institute (EPHI), Addis Ababa, Ethiopia, and maintained in the animal house at School of Pharmacy, Addis Ababa University. All animals were housed in an air-conditioned room and allowed to acclimatize for 1 week before the study. The animals were kept at room temperature and exposed to a 12 hour light/dark cycle. They had free access to commercial pellet diet and clean drinking water. All procedures and techniques used in this study were in accordance with the guide for care and use of laboratory animals [11].

Grouping and dosing: the animals were randomly assigned into five groups, each group consisting of six mice (*n* = 6). The first group was assigned with negative controls treated with the distilled water. The second group was assigned as positive control and treated with standard drugs, sodium valproate 200 mg/kg (SV200) for the PTZ-induced seizure test, and phenytoin 25 mg/kg (PTN25) for the MES-induced seizure test. The rest of three groups received increasing doses of extracts (100, 200, and 400 mg/kg).

Acute oral toxicity study: the acute toxicity of *Pentas schimperiana* (A. Rich.) Vatke root bark was determined based on Organization of Economic Cooperation and Development (OECD) guideline no. 425. It was observed that the test extract was not mortal at 2000 mg/kg dose. As per OECD 425 guideline, 1/10th of the limit dose 2000 mg/kg was taken as a middose (200 mg/kg) after which half and doubling of the middose were selected as minimum and maximum doses (100 mg/kg and 400 mg/kg, respectively) for the study [12].

### 2.5. Anticonvulsant Activity Tests

Pentylenetetrazole- (PTZ-) induced seizure test is considered as indicative of anticonvulsant activity of drugs against absence and myoclonic seizures. PTZ produces generalized synchronized clonic movements, which are superseded by tonic convulsion characterized by flexion of limbs followed by extension. For this experiment, the method described by [13] was used. The mice received 100, 200, and 400 mg doses of extract, sodium valproate, and vehicle through the oral route. After 60 min of these treatments, PTZ at 85 mg/kg in distilled water was injected through subcutaneous route for each mouse. Each mouse was placed into a transparent cage and observed for convulsive behavior for 30 min by using a video recorder. The clonic seizure of forelimb and hind limb were taken as an end point in this test. The percentage protection of clonic seizure, percentage protection of mortality, and mean latency to clonic convulsions (*s*) were noted and compared with that of vehicle control.

(1) $$\begin{array}{c} \text{\% protection from Cs}=\left(\frac{\text{No clonic seizure in control}-\text{no clonic seizure in test}}{\text{No clonic seizure in control}}\right)*100,\\ \text{\% protection from mortality}=\left(\frac{\text{No death in control}-\text{no death in test}}{\text{No death in control}}\right)*100. \end{array}$$

Maximal electroshock- (MES-) induced seizures test: protection against electroshock-induced seizures in mice was used as an indication for compounds, which may prove effective in generalized seizures of the tonic-clonic (grand mal) type. For this study, a protocol developed by [14] was used.

In brief, mice in different groups received 100, 200, and 400 mg doses of extract, phenytoin, and vehicle through the oral route.

After 60 min of these treatments, the mice received maximal electric shocks of 50 mA for 0.2s through ear-clip electrodes by using an electroconvulsiometer (Rolex Ambala, India). Following stimulus application, the vehicle-treated mice were shown an immediate severe tonic seizure with the maximal extension of the anterior and posterior legs.

The body becomes stiffened and lasted for 12–16 s; at the end of this tonic phase, variable phase of clonic seizures was started, characterized by paddling movements of the hind limbs and shaking of the body; 20–50 s later, the animals were maintained in their upright position and start moving around, apparently recovering their normal behavior. The mice were observed closely for 2 min by using a video recorder.

The reduction in the duration of tonic hind limb extension (THLE) compared to the control group was considered as evidence for the presence of anticonvulsant activity.

(2) $$\begin{array}{c} \text{\% reduction in dr}.\text{ of THLE}=\left(\frac{\text{Mean dr}.\text{ of THLE in control}-\text{mean dr}.\text{ of THLE in test}}{\text{Mean dr}.\text{ of THLE in control}}\right)*100. \end{array}$$

### 2.6. Preliminary Phytochemical Analysis

The extract of the root bark of *Pentas schimperiana* (A. Rich.) Vatke was subjected to phytochemical screening tests for the detection of various constituents [15].

### 2.7. Statistical Analysis

Results were analyzed using windows Statistical Package for Social Science (SPSS), version 21. All experimental data were expressed as mean values ± standard error of mean (SEM) and analyzed through a one-way ANOVA followed by the post hoc test (Tukey test) for multiple comparisons of the mean differences and responses of different extracts with controls. The analysis was performed with 95% confidence interval, and the significance was set at *p* < 0.05.

## 3. Results

**Figure 1:**
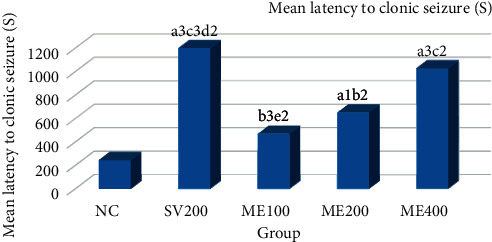
The anticonvulsant effect of 80% methanol extract of *Pentas schimperiana* (A. Rich.) Vatke root bark in PTZ-induced seizure.

**Figure 2:**
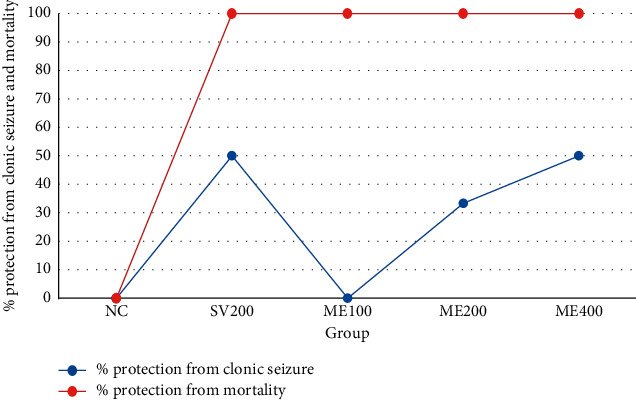
The percentage protection from mortality and clonic seizure of 80% methanol extract of *Pentas schimperiana* (A. Rich.) Vatke root bark in PTZ-induced seizure.

The anticonvulsant effects of *Pentas schimperiana* (A. Rich.) Vatke by ME in the PTZ-induced seizure test are shown in Figure 1. The result showed that mean latency time to clonic seizure was significantly increased at doses of ME400 (*p* < 0.001) and ME200 (*p* < 0.05) when compared with negative control. Maximum percentage (66.67%) protection from mortality and clonic seizure is also achieved by ME400 than other doses of ME (Figure 2).

Anticonvulsant effects of PTZ-induced seizure in solvent fractions of the plant showed that (Table 1) all doses of BF-tested groups significantly (*p* < 0.001) increased in the latency time of clonic seizure compared to negative control. Maximum percentage (50.00%) protection from mortality and clonic seizure was achieved by BF400 than other doses of BF. In addition to BF doses, a significant effect on increment of mean latency time to clonic seizure had been observed by doses of CF400 (*p* < 0.001), CF200 (*p* < 0.001), and CF100 (*p* < 0.05).

**Figure 3:**
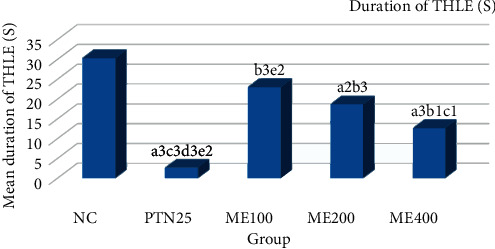
The anticonvulsant effect of 80% methanol extract of *Pentas schimperiana* (A. Rich.) Vatke *e* root bark in MES-induced seizure.

**Figure 4:**
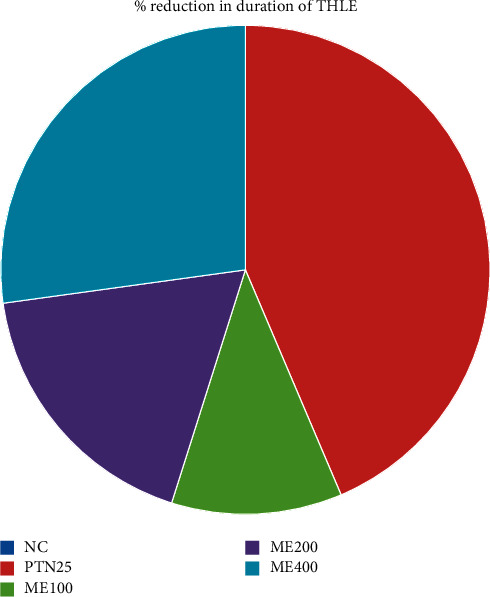
Percentage reduction in duration of THLE of 80% methanol extract of *Pentas schimperiana* (A. Rich.) Vatke root bark in MES-induced seizure.

The effect of *Pentas schimperiana* (A. Rich.) Vatke 80% methanol extraction MES-induced seizure is shown in Figure 3. The study showed that the dose of the ME400 group significantly (*p* < 0.001) reduced the mean duration of THLE compared with negative control. The percentage reduction in the duration of THLE by ME400 was 58.23%, which was greater than ME100 and ME200 but lower than that of PTN25 (Figure 4).

The anticonvulsant activity test of the aqueous, butanol, and chloroform fraction of *Pentas schimperiana* (A. Rich.) Vatke is also further evaluated by using the MES test (Table 2). No statistically significant difference was observed between solvent fractions doses and control in reduction mean duration of THLE, except doses of BF200 (*p* < 0.05) and BF400 (*p* < 0.001). BF400 also showed the maximum percentage reduction (54.40%) in the duration of THLE than the other fractions. On the other hand, CF-tested doses exhibited slight reduction in the duration of THLE.

Preliminary phytochemical analysis showed that the *Pentas schimperiana* (A. Rich.) Vatke root bark extract contained tannins, alkaloids, terpenoids, flavonoids, steroids, phenolic compounds, and proteins.

## 4. Discussion

This study was conducted to evaluate the anticonvulsant activity of 80% methanol extract and solvent fraction of *Pentas schimperiana* (A. Rich.) Vatke root bark plant. PTZ- and MES-induced seizure tests, which are acute seizure models, were used in this study. The drugs which antagonize the PTZ-induced convulsions are known to be effective in petitmal epilepsy. PTZ is known to possess GABA antagonistic activity [16]. The MES-induced seizure test in mice primarily indicates the compounds which are effective in grand mal epilepsy. The tonic extension of the hind limb evoked by electrical stimuli is suppressed by antiepileptics. Antiepileptic drugs that block MES-induced seizure are known to act by blocking the seizure spread [17]. The models have been developed >60 years ago, and there were standard starting in early stages of many AED screening programs [18]. This is used because it is easy to perform, time- and cost-efficient, and shows good reproducibility between laboratories, well validated with several AEDs and predictive of clinical activity [19].

In the PTZ-induced seizure model study, ME400 dose of crude extract showed significant anticonvulsant activity against PTZ-induced clonic seizure. This might be due to possible localization of active ingredients and presence bioactive secondary metabolites contained in the plant. From solvent fractions against PTZ-induced seizure, butanol fraction had exerted superior increment in the latency time to onset of clonic seizure and percentage protection from mortality than other fraction. However, the anticonvulsant effect was lower than that of crude extract.

The highest effect might be due to the presence of most bioactive secondary metabolites, such as flavonoid, saponins, phenols, terpenoids, and tannins, and appeared to be responsible for the observed anticonvulsant activity of this fraction. Activity reduction in the aqueous fractions might be due to the absence of most bioactive secondary metabolites.

Chloroform fraction was found to have the second highest anticonvulsant activities from fractions in the PTZ test. The absence of flavonoid and other metabolites might be reason for this fraction to have lower activity than a butanol fraction.

PTZ-induced seizures are abolished by agents that act by reducing T-type calcium channel and/or enhance GABAergic neurotransmission [20]. It has been found that drugs which inhibit PTZ-induced seizures are generally effective against myoclonic and absence seizures [15]. Therefore, it is possible to say that the anticonvulsant effects shown by study plant in the PTZ-induced seizure test might be due to inhibition of T-type calcium channel or enhancement of GABAergic neurotransmission. However, it needs further study.

In the MES-induced seizure model, crude extract, mainly ME400 dose, significantly reduced the mean duration of THLE. This indicates that the higher dose, ME400, could be taken as the maximum effective dose relative to ME200 and ME100 doses. This might be due to the presence of good concentrations of active compounds. Butanol fraction had exerted a higher reduction in the duration of THLE than aqueous and chloroform fraction. Some of AEDs such as phenytoin, carbamazepine, lamotrigine, and valproate are effective against this seizure model act by blocking sodium channels, thereby stabilizing the inactive state of sodium channels, thus reducing high frequency firing of action potentials [21].

In fact, these drugs have multiple mechanisms of actions and are effective in both animal models [20, 21]. Therefore, the higher dose crude extract and butanol fraction have anticonvulsant activity; this might be due to phytoconstituents present in both extracts that exert their broad-spectrum anticonvulsant activity such as blocking Na^+^ channel and/or enhancement of GABAergic neurotransmission against generalized tonic-clonic seizures.

In contrast to the PTZ model, the chloroform fraction did not demonstrate anticonvulsant activity against MES-induced seizure in mice. The absence of the anticonvulsant effect was probably lack of bioactive secondary metabolite such as flavonoid and saponins in this fraction. As mentioned earlier, the butanol fraction containing these metabolites showed significant anticonvulsant activity in MES.

Preliminary phytochemical analysis of the *Pentas schimperiana* (A. Rich.) Vatke revealed the presence of tannins, alkaloids, terpenoids, flavonoids, steroids, phenolic compounds, and proteins.

Based on the present knowledge of the chemical constituents, it is not possible to attribute with certainty the detected active principle/s for its anticonvulsant activity. However, several flavonoids could act as benzodiazepine-like molecules in the central nervous system and modulate GABA-mediated chloride channels in animal models of anxiety, sedation, and convulsion. Certain terpenoids and steroids are reported to possess anticonvulsant activity in MES and PTZ experimental seizure models [22].

## 5. Conclusion

The overall investigation of *Pentas schimperiana* (A. Rich.) Vatke root bark extract for its anticonvulsant activity produced a result supporting the traditional claim. However, further studies are required to isolate, identify, characterize, and elucidate the structure of specific phytomolecules responsible for observed biological activities in this study and their precise mechanism(s) of action.

## Figures and Tables

**Table 1:** Anticonvulsant effect of solvent fraction of *Pentas schimperiana* (A. Rich.) Vatke root bark in PTZ-induced seizure.

Group	% protection from clonic seizure	% protection from mortality	Mean latency to clonic seizure (S)
NC	—	—	246.17 ± 45.13
SV200	100	100	1200.00 ± 0.00^a3c2^
AF100	—	—	700.00 ± 73.76^a1b2^
AF200	—	—	850.00 ± 93.49^a3^
AF400	—	16.67	870.00 ± 96.44^a3^
NC	—	—	246.17 ± 45.13
SV200	100	100	1200.00 ± 0.00^a3^
BF100	—	16.67	1000.00 ± 100.00^a3^
BF200	33.33	33.33	820.00 ± 124.58^a3^
BF400	50.00	50.00	1010.00 ± 98.49^a3^
NC	—	—	246.17 ± 45.13
SV200	100	100	1200.00 ± 0.00^a3c2^
CF100	—	16.67	660.00 ± 57.97^a1b2^
CF200	33.33	50.00	880.00 ± 110.27^a3^
CF400	33.33	50.00	960.00 ± 81.98^a3^

Data are expressed as mean ± SEM. *n* = 6 mice. ^a^Compared to NC; ^b^compared to SV200; ^c^compared to 100 mg/kg; ^d^compared to 200 mg/kg; ^e^compared to 400 mg/kg. ^1^*p* < 0.05, ^2^*p* < 0.01, ^3^*p* < 0.001. NC, group treated with distilled water (10 ml/kg); SV200, sodium valproate (200 mg/kg); AF, aqueous fraction of *Pentas schimperiana* (A. Rich.) Vatke; BF, butanol fraction of *Pentas schimperiana* (A. Rich.) Vatke; CF, chloroform fraction of *Pentas schimperiana* (A. Rich.) Vatke. Numbers refer to dose in mg/kg.

**Table 2:** Anticonvulsant effect of solvent fraction of *Pentas schimperiana* (A. Rich.) Vatke root bark in MES-induced seizure.

Group	% reduction in duration of THLE	Mean duration of THLE (S)
NC	—	30.33 ± 4.12
PTN25	93.41	2.00 ± 1.26^a3c3d3e3^
AF100	1.65	29.83 ± 1.45^b3^
AF200	7.68	28.00 ± 1.39^b3^
AF400	9.33	27.50 ± 1.77^b3^
NC	—	30.33 ± 4.12
PTN25	93.41	2.00 ± 1.26^a3c3d3e3^
BF100	18.67	24.67 ± 1.86^b3e2^
BF200	35.15	19.67 ± 2.26^a1b3^
BF400	54.40	13.83 ± 1.78^a3b2c2^
NC	—	30.33 ± 4.12
PTN25	93.41	2.00 ± 1.26^a3c3d3e3^
CF100	1.65	29.83 ± 1.70^b3^
CF200	5.47	28.67 ± 1.58^b3^
CF400	7.12	28.86 ± 1.78^b3^

Data are expressed as mean ± SEM. *n* = 6 mice. ^a^Compared to NC; ^b^compared to PTN25; ^c^compared to 100 mg/kg; ^d^compared to 200 mg/kg; ^e^compared to 400 mg/kg.^1^*p* < 0.05, ^2^*p* < 0.01, ^3^*p* < 0.001. NC, group treated with distilled water (10 ml/kg), PTN25, phenytoin (25 mg/kg); THLE, tonic hind limb extension; AF, aqueous fraction of *Pentas schimperiana* (A. Rich.) Vatke; BF, butanol fraction of *Pentas schimperiana* (A. Rich.) Vatke; CF, chloroform fraction of *Pentas schimperiana* (A. Rich.) Vatke. Numbers refer to dose in mg/kg.

## Data Availability

The data used to support the findings of this study are available from the corresponding author upon request.

## Abbreviations

AEDs: Antiepileptic drugs
AF: Aqueous fraction
ANOVA: Analysis of variance
BF: Butanol fraction
CF: Chloroform fraction
Cm: Centimeters
EPHI: Ethiopian Public Health Institute
GABA: *γ*-Aminobutyric acid
GluRs: Glutamate receptors
H: Hour
HCl: Hydrochloric acid
IP: Intraperitoneal
K: Potassium
Kg: Kilogram
M: Meter
ME: Methanol extract
MES: Maximal electrical shock
mA: Milliampere
Min: Minute
Mg: Milligram
S: Second
OECD: Organization of Economic Cooperation and Development
PTZ: Pentylenetetrazole
SEM: Standard error of mean
SNNPR: Southern Nations, Nationalities, and Peoples Region
SPSS: Statistical Package for Social Science
THLE: Tonic hind limb extension.
